# Identification of sialylated glycoproteins from metabolically oligosaccharide engineered pancreatic cells

**DOI:** 10.1186/s12014-015-9083-8

**Published:** 2015-04-11

**Authors:** Yuan Tian, Ruben T Almaraz, Caitlin H Choi, Qing Kay Li, Christopher Saeui, Danni Li, Punit Shah, Rahul Bhattacharya, Kevin J Yarema, Hui Zhang

**Affiliations:** Department of Pathology, Johns Hopkins University, 400 N. Broadway, Room 4011, Baltimore, MD 21287 USA; Department of Biomedical Engineering and the Translational Tissue Engineering Center, Johns Hopkins University School of Medicine, Baltimore, MD USA

**Keywords:** Sialylated glycoproteins, Metabolic oligosaccharide engineering, Pancreatic cancer cells

## Abstract

In this study, we investigated the use of metabolic oligosaccharide engineering and bio-orthogonal ligation reactions combined with lectin microarray and mass spectrometry to analyze sialoglycoproteins in the SW1990 human pancreatic cancer line. Specifically, cells were treated with the azido *N*-acetylmannosamine analog, 1,3,4-Bu_3_ManNAz, to label sialoglycoproteins with azide-modified sialic acids. The metabolically labeled sialoglyproteins were then biotinylated via the Staudinger ligation, and sialoglycopeptides containing azido-sialic acid glycans were immobilized to a solid support. The peptides linked to metabolically labeled sialylated glycans were then released from sialoglycopeptides and analyzed by mass spectrometry; in parallel, the glycans from azido-sialoglycoproteins were characterized by lectin microarrays. This method identified 75 unique N-glycosite-containing peptides from 55 different metabolically labeled sialoglycoproteins of which 42 were previously linked to cancer in the literature. A comparison of two of these glycoproteins, LAMP1 and ORP150, in histological tumor samples showed overexpression of these proteins in the cancerous tissue demonstrating that our approach constitutes a viable strategy to identify and discover sialoglycoproteins associated with cancer, which can serve as biomarkers for cancer diagnosis or targets for therapy.

**Electronic supplementary material:**

The online version of this article (doi:10.1186/s12014-015-9083-8) contains supplementary material, which is available to authorized users.

## Introduction

Altered patterns of glycosylation are a universal feature of cancer with sialic acids – which are a group of unusual amino-modified acidic sugars ubiquitously displayed on the outer ends of mammalian glycan chains [1] – playing an especially prominent role as tumor-associated carbohydrate antigens (TACAs) [2,3]. TACAs, in general, are involved in many biological and pathological processes, such as regulating cellular and molecular interactions by either masking recognition sites or serving as recognition determinants [4]. More specifically, altered sialylation of tumor cell surfaces is associated with several critical malignant properties that include invasiveness and metastatic potential [5-7]. Cell surface sialylation is controlled by several factors, which include regulation of metabolic flux of ManNAc into the sialic biosynthetic pathway [8,9], changes in the expression and enzymatic activities of sialyltransferases and sialidases, and the availability of candidate penultimate glycan termini on glycoproteins that serve as acceptor sites for sialylation [5,10].

An interesting observation made about two decades ago was that cancer cells expressed the non-human “Neu5Gc” form of sialic acid at much higher levels than normal tissue [11]. The accumulation of Neu5Gc in tumors was attributed to scavenge of dietary Neu5Gc [12], which was ultimately biosynthetically incorporated into sialylated TACAs at much higher levels than into the sialoglycans of healthy cells. Building on the observation, the enhanced ability of tumors to over-express types of exogenously-supplied sialic acid other than the canonical human “Neu5Ac” form, has been proposed as a new approach to cancer therapy with Neu5Prop and Neu5Phenyl analogs being evaluated for immunotherapy purposes [13]. Similarly, sialic acid precursors with chemical functional groups such as the ketone [14] or azide [15] not normally found in mammalian sugars have been applied to the preferential delivery of diagnostic or therapeutic agents to tumors. The emerging success of these strategies is evident in the recent *in vivo* demonstration that azido-modified sialic acids derived from corresponding ManNAc metabolic precursors are highly expressed in tumors compared to healthy tissues [16].

While a variety of high-throughput approaches have been used to study sialylated glycoproteins [17], which include lectin affinity [6,10,18], titanium dioxide affinity chromatography [19], strong cation exchange columns [20], conditional hydrazide chemistry [7,18,21-24], or the combination of diagonal chromatographic technology and neuraminidase treatments [25], the above-mentioned metabolic oligosaccharide engineering strategy is an emerging chemical method to analyze glycoproteins that has become increasingly used in the past decade [15,26]. In this approach, an unnatural monosaccharide with a bio-orthogonal functional group can be introduced into the biosynthetic pathways of living cells and incorporated into cellular glycoproteins. The bio-orthogonal functional group can function as a chemical handle and be tagged with complementary reactive partners via covalent ligation reactions. An important feature of metabolic oligosaccharide engineering is that the labeling of glycans can occur in both living cells and animals and the method can be used to manipulate and study glycosylation events [27]. Accordingly, this method is becoming valuable for glycoproteomics studies in both eukaryotic and bacterial systems with investigation of cancer a particularly active area of research [27-32].

Based on the selective *in vivo* incorporation of exogenously supplied monosaccharides into tumors, as discussed above, we reasoned that a metabolic oligosaccharide engineering approach could be used to meet a present need in understanding cancer, which is to more thoroughly characterize the role of sialoglycoproteins in tumor progression. As a caveat, the selective incorporation of non-natural sialic acids into tumors could simply be a consequence of the increased metabolic activity of cancer cells compared to most normal cells found in the body. However, a previous study where we treated the human SW1990 pancreatic cancer cell line with the “high flux” 1,3,4-O-Bu_3_ManNAc analog that increases levels of natural sialic acid , showed that increased sialylation did not occur evenly across all glycoproteins. Instead, certain glycoproteins did not experience a measurable increase in sialylation while important cancer-related markers including CD44 and integrin α6 [9] as well as EGFR [33] experienced increased levels of sialic acid of 2-fold or more. Increased levels of *natural* sialic acid, however, hold less potential to manipulate or study the system compared to a similar approach where *non-natural* sialosides are metabolically installed into cancer-associated glycans. Therefore, in the present study, we investigated whether treatment of SW1990 cells with the non-natural azido-modified sialic acid precursor 1,3,4-O-Bu_3_ManNAz [34] would similarly preferentially label cancer-associated sialoglycans and, by doing so, provide a method for the discovery, isolation, and study of this important class of tumor-promoting molecules.

**Figure 1:**
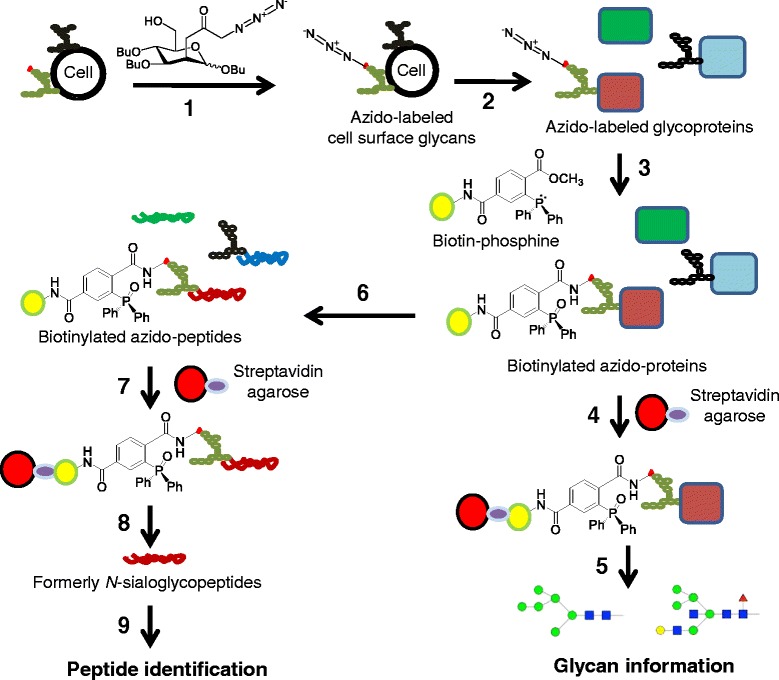
Strategy for analyzing azide-modified sialoglycoproteins. The strategy used to analyze the samples includes multiple steps as follows: **1)** Cells were metabolically labeled using 1,3,4-*O*-Bu_3_ManNAz. **2)** Proteins were extracted using RIPA buffer at which point samples were divided with one set of aliquots used for steps 4 and 5 and another set of aliquots used for steps 6 through 9. **3)** Azide-labeled proteins were biotinylated using through the Staudinger reaction using biotin-PEG_3_-phosphine and excess reagent was removed by protein precipitation. **4)** Biotinlabeled, azide-modified proteins were purified using monomeric avidin agarose. **5)** Glycan profiles of biotin-labeled, azide-modified proteins were determined by lectin microarray analysis. **6)** Proteins were trypsin digested after biotinylation. **7)** Biotin-labeled peptides were coupled to streptavidin agarose. **8)** PNGase F was used to release the formerly *N*-glycosylated peptide from the agarose beads. **9)** The released peptides were analyzed by LC-MS.

To experimentally verify the incorporation of azido-sialic acids into cancer-associated glycans, we treated SW1990 cells with 1,3,4-O-Bu_3_ManNAz and adapted methods previously used in our groups (as outlined in Figure 1) to extract and enrich azido-labeled glycopeptides and glycoproteins based on the modified Staudinger reaction first reported by the Bertozzi group [15]. Once enriched, the samples were analyzed by mass spectrometry to determine the identity of proteins labeled by azido-modified glycans and by lectin analysis to gain a sense of the composition of the actual glycan structures. Once this knowledge was in hand, we analyzed clinical samples to verify that proteins that were identified were selectively associated with cancer compared to healthy tissue.

## Results

### Incorporation of azido-groups into cellular glycoconjugates

**Figure 2:**
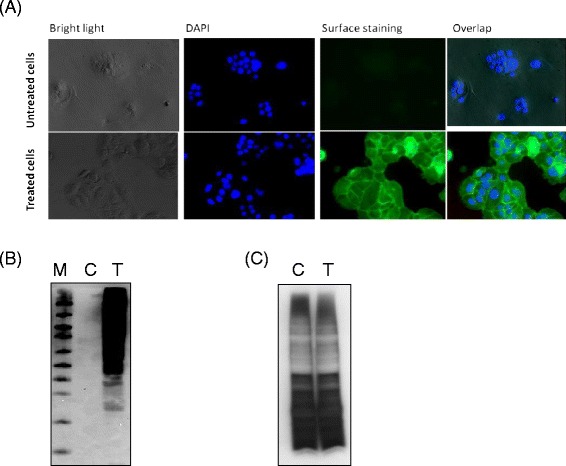
Assessment of metabolic incorporation of azido-modified sialic acids into cellular glycans. (**A**) Cell surface labeling of SW1990 cells incubated with 1,3,4-*O*-Bu_3_ManNAz for two days. Labeling of the resulting surface azido-glycoconjugates, indicated by green fluorescence, was achieved by click chemistry with an Alexa488 fluorophore and the cells were counterstained with DAPI (blue fluorescence) as a nucleus marker. (**B**) Proteins were harvested from untreated control (denoted by “C”) and treated (denoted by “T”) SW1990 cells and the specific biotinylation of azide-modified proteins was assessed by Western blots (“M” denotes molecular weight markers). (**C**) Silver staining of PAGE-separated proteins from control (denoted “C”) or treated (denoted “T”) SW1990 cells verified that similar amounts of protein were analyzed in both cases.

Metabolic oligosaccharide engineering-based incorporation of 1,3,4-*O*-Bu_3_ManNAz into the glycoconjugates of various cell lines including SW1990 was recently investigated [34], suggesting that 50 μM of analog was an adequate concentration to robustly label sialoglycans without inducing any noticeable cytotoxicity, growth inhibition, or other “off-target” effects. The series of experiments shown in Figure 2 confirmed that 50 μM was an appropriate analog concentration to conduct the metabolic labeling reported in the current study. In particular, SW1990 cells were treated with 50 μM 1,3,4-*O*-Bu_3_ManNAz and then labeled by adding Click-iT reaction cocktail to conjugate fluorescent dyes to the azide groups of sialic acids. Fluorescent microscopy showed strong azide-derived signal in 1,3,4-*O*-Bu_3_ManNAz treated cells and virtually no background signal in the untreated controls (Figure 2A). In complementary experiments designed to verify the metabolic incorporation of azido analogs into the glycoconjugates of SW1990 cells, proteins were extracted from treated or untreated cell lysates, biotinylated via a modification of the Staudinger ligation reaction [15], separated by electrophoresis, and evaluated using HRP-streptavidin (Figure 2B). The data showed the detection of numerous biotinylated proteins of a broad ranges of molecular weights in lysates derived from 1,3,4-*O*-Bu_3_ManNAz treated cells but virtually no signal from lysates labeled in a similar manner but derived from the untreated control cells. Silver staining (Figure 2C) showed that similar amounts of proteins were loaded for each blot, confirming that the biotin tag was specifically added only to azido-sugars present in the glycans of analog treated cells via the intended bioorthogonal ligation reaction between the azide and phosphine groups.

### Lectin microarray analysis

**Figure 3:**
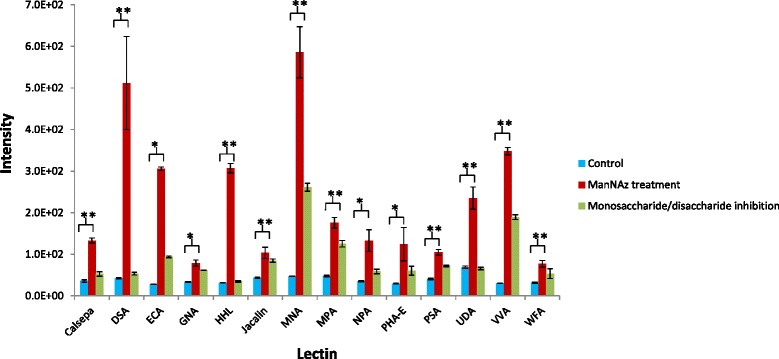
Glycan analysis of azido-modified glycoproteins. Glycan profiles of azido-modified glycoproteins analyzed by lectin microarray. Fourteen lectins showed significant binding to treated cells compared to control cells (*p < 0.05 and **p < 0.01). The lectin binding signals were inhibited by the appropriate mono- and disaccharides.

To investigate the glycosylation patterns on azido-sialic acid containing glycoproteins in pancreatic cancer cells, our previously developed lectin microarray [35] was used to bind biotinylated glycoproteins (Figure 1, Steps 4 and 5) that had first been affinity purified by monomeric avidin agarose resin from 1,3,4-*O*-Bu_3_ManNAz-treated and control cells (Figure 1, Steps 1–3). Streptavidin-conjugated Dylight 549 was used to detect the biotinylated azido-sialoglycoproteins on lectin microarray. No detectable binding signal above background was obtained from samples from the control cells without ManNAz analog treatment (Figure 3). In contrast, glycoproteins obtained from treated cells showed high binding intensity to 10 of the 38 lectins on the array with a significant difference of *p* < 0.01 above the background while binding to four additional lectins showed lower intensity that was nonetheless significantly above background levels (*p* < 0.05). Binding to the lectins was specifically inhibited by a monosaccharide or a disaccharide that competes the binding to the same lectin, which confirmed the binding signal was specific (Figure 3). Lectins with significant binding signal were DSA, PHA-E, PSA, WFA, Jacalin, MPA, ECA, UDA, GNA, HHL, NPA, Calsepa, MNA-G, and VVA (Figure 3 and Table 1). Based on the glycan-binding specificity of these lectins, among the *N*-type glycans, the azide-labeled sialoglycoproteins contained α-1-6 core fucoses (PSA), Galβ1-4GlcNAc structures (DSA and PHAE), and high mannose (NPA, GNA, and HHL) (Table 1 and Additional file 1: Table S1). For *O*-type glycans, the glycan structure contained Galβ1-3GalNAc (Jacalin and MPA) and GalNAcβ1-4GlcNAc (WFA) (Table 1 and Additional file 1: Table S1). The most intensive binding signal was detected in DSA and NPA (Figure 3).

**Table 1:** **Lectins showed specific binding signal to** **azide-modified glycoproteins**

**Lectin**	**Full name**	Specificity*	***p*** **value**
DSA	*Datura stramonium*	(GlcNAcβ1-4)*n*, Galβ1-4GlcNAc	<0.01
PHA-E	*Phaseolus vulgaris E*	Complex-type *N*-glycans with outer Gal and bisecting GlcNAc	<0.05
PSA	*Pisum sativum*	Fucα1-6GlcNAc, α-D-Glc, α-D-Man	<0.01
WFA	*Wisteria floribunda*	GalNAcβ1-4GlcNAc, Galβ1-3(-6)GalNAc	<0.01
Jacalin	*Artocarpas integliforia*	Galβ1-3GalNAc, GalNAc	<0.01
MPA	*Maclura pomifera*	Galβ1-3GalNAc, GalNAc	<0.01
ECA	*Erythrina cristagalli*	Galβ1-4GlcNAc (Terminal)	<0.05
UDA	*Urtica dioica*	GlcNAcβ1-4GlcNAc, Mixture of Man5 to Man9	<0.01
GNA	*Galanthus nivalis*	High-Man, Manα1-3Man	<0.05
HHL	*Hippeastrum Hybrid*	High-Man, Manα1-3Man, Manα1-6Man	<0.01
NPA	*Narcissus pseudonarcissus*	High-Man, Manα1-6Man	<0.05
Calsepa	*Calystegia sepium*	Man, maltose	<0.01
MNA-G	*Morus nigra G*	Man	<0.01
VVA	*Vicia villosa*	α-Linked terminal GalNAc, GalNAcα1-3Gal	<0.01

*Lectin frontier Database; http://riodb.ibase.aist.go.jp/rcmg/glycodb/LectinSearch and references [36,37].

### Identification and characterization of azide-labeled sialoglycoproteins by LC-MS/MS

By using the strategy outlined in Figure 1, Steps 6 to 9, 75 unique formerly azido-sialic acid modified glycopeptides were identified in samples obtained from SW1990 cells treated with 1,3,4-*O*-Bu_3_ManNAz analog, representing 55 different glycoproteins (Table 2 and Additional file 2: Table S2). The MS/MS annotation of all the identified peptides is provided in Additional file 3: Table S3. No glycoproteins were identified from cells that were not treated with 1,3,4-O-Bu_3_ManNAc, indicating the specific enrichment and release of azido-sialic acid containing sialoglycoproteins. Approximately 17% of the proteins were identified by two or more unique glycopeptides, e.g., galectin-3-binding protein (GAL3BP) was identified by 5 glycopeptides, and lysosome-associated membrane glycoprotein 1 (LAMP1) identified by 4 glycopeptides.

**Table 2:** **Identification of azido-sialic acid modified** ***N*** **-linked sialoglycoproteins**

**Protein group accessions**	**Protein name**	**Gene symbol**
167466198	Intercellular adhesion molecule 1	ICAM1
296010912	Tissue factor	CD142
7669492	Glyceraldehyde-3-phosphate dehydrogenase	GAPD
4503143	Cathepsin D	CTSD
4505467	5′-nucleotidase isoform 1	NT5E
4507483	Thrombomodulin	THBD
9845238	UDP-GlcNAc:βGal β-1,3-N-acetylglucosaminyltransferase 2	B3GNT2
223468595	Integrin α-V	Itgav
20150648	Chain A,Human Dipeptidyl Peptidase I (Cathepsin C)	CTSC
19743823	Integrin β-1	Itgb1
29550838	Golgi membrane protein 1	GOLM1
53829379	Urokinase plasminogen activator surface receptor	PLAUR
94962177	Activated leukocyte cell adhesion molecule variant 1	Alcam
38327634	ATP-dependent RNA helicase DDX18	DDX18
51247095	Chain T, Tissue Factor-Factor Viia Complex	
116534898	Desmoglein-2	DSG2
4505061	Cation-dependent mannose-6-phosphate receptor	M6PR
22202611	Carboxypeptidase D	cpd
85544358	Chain C, A Minimal Gas6-Axl Complex	
227430301	CD109 antigen	CD109
4507509	Metalloproteinase inhibitor 1	TIMP1
189458817	Transferrin receptor protein 1	TFRC
4959370	Radical fringe	RFNG
91199546	CD63 antigen	CD63
5031863	Galectin-3-binding protein	LGALS3BP
6014587	Mesothelin/megakaryocyte potentiating factor	MSLN
10863927	Peptidyl-prolyl cis-trans isomerase A	PPIAL3
149363636	Plexin-B2	plxnb2
157266292	Intestinal-type alkaline phosphatase	ALPI
294660768	MHC class I polypeptide-related sequence A (MICA*00801)	MICA
27754771	Protocadherin-1	PCDH1
222831610	Choline transporter-like protein 2	SLC44A2
47419930	Chondroitin sulfate proteoglycan 4	cspg4
32967311	Ephrin type-A receptor 2	EPHA2
4758950	Peptidyl-prolyl cis-trans isomerase B	ppiB
112380628	Lysosome-associated membrane glycoprotein 1	lamp1
4504957	Lysosome-associated membrane glycoprotein 2	lamp2
38569398	Integrin α-10	ITGA10
4506113	Major prion protein	PRNP
21536337	Myelin protein zero-like protein 2	MPZL2
187828910	CD59 glycoprotein	CD59
10092665	Sushi domain-containing protein 2	Susd2
68163411	CD166 antigen	Alcam
190194386	Transmembrane 9 superfamily member 3	TM9SF3
180225	Carcinoembryonic antigen	CEACAM5
270483821	Tetraspanin-3	TSPAN3
7657373	Tetraspanin-13	TSPAN13
61744483	4F2 cell-surface antigen heavy chain	SLC3A2
24308201	Adipocyte plasma membrane-associated protein	C20orf3
5542165	Chain A, Human Platelet Profilin Complexed With An L-Pro10- Iodotyrosine Peptide	
5453832	Hypoxia up-regulated protein 1	hyou1
310125063	HLA class I histocompatibility antigen, A-43 α chain-li	
148728160	Receptor-type tyrosine-protein phosphatase eta	PTPRJ
166235138	Seizure 6-like protein 2 isoform 3	sez6l2
166235140	Seizure 6-like protein 2 isoform 4	sez6l2

### Analysis of metabolically labeled sialoglycoproteins labeled with azido-sialoglycans

To gain insight into the biological activities of the glycoproteins that had been isolated and identified from the 1,3,4-*O*-Bu_3_ManNAz treated SW1990 cells, GO analysis was performed [38]. The analysis of cellular component shows that the majority of identified proteins (83%) are extracellular proteins, including cell membrane proteins and secreted proteins. The analysis of molecular function shows that these glycoproteins play important roles in cell signal transduction and cell-cell interaction including catalytic activity (32.7%), receptor activity (29.1%), binding (25.5%), enzyme regulator activity (5.5%), transporter activity (5.5%), and ion channel activity (1.8%). The analysis of biological process again reveals a broad range of activities for the identified proteins including cellular process (15.0%), metabolic process (14.4%), cell communication (12.4%), immune system process (11.8%), cell adhesion (11.1%), response to stimulus (8.5%), and developmental process (6.5%). Investigation of the identified sialoglycoproteins using previous reported glycoproteins showed that among the 55 identified sialoglycoproteins labeled by sialic acid analog, 42 proteins have documented correlation with cancer (Additional file 2: Table S2), including the 4F2 cell-surface antigen, CD 109 antigen, integrin β1, and carboxypeptidase D [39-42]. In addition, fifteen of the proteins have been specifically linked to pancreatic cancer (Additional file 2: Table S2), including cathepsin D, glyceraldehyde-3-phosphate dehydrogenase, intercellular adhesion molecule 1, mesothelin, and tissue factor [43-47].

### Implications of cancer-specific sialoglycoproteins identified from metabolically glycoengineered cells

To test whether the glycoproteins identified from our metabolic oligosaccharide engineering approach have potential clinical significance (e.g., as biomarkers), immunohistological analysis was performed on cancerous and non-cancerous pancreatic tissue. Lysosome-associated membrane glycoprotein 1(LAMP1) and hypoxia up-regulated protein 1 (ORP 150) were chosen for analysis in this regard because they previously have not been associated with pancreatic cancer. LAMP1 is a glycoprotein usually expressed in endosomes and lysosomal substructures of cells with less expression on the plasma membrane [48,49]. Elevated expression of LAMP1 at the cell surface has been reported in metastatic tumor cells although no link with pancreatic cancer has been reported [50]. Similarly, no connection with pancreatic cancer yet has been reported for ORP 150, a protein that plays a role in protein folding and secretion in the ER and hypoxia-induced cellular perturbation and is up-regulated in certain tumors, especially in breast cancer [51-55].

**Figure 4:**
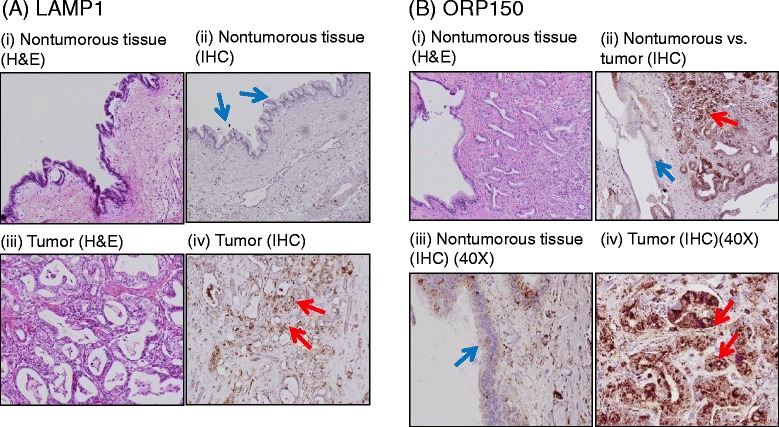
Verification of protein expression in pancreatic cancer and matched nontumorous pancreas tissues using IHC. (**A**) Increased expression of LAMP1 in pancreatic tumor: (i) H&E staining of nontumorous pancreas duct, (ii) low expression of LAMP in matched nontumorous pancreas ductal cells, (iii) H&E staining of pancreatic tumors, and (iv) overexpression of LAMP in pancreatic tumor. (**B**) Increased expression of ORP150 in pancreatic cancer tissue as compared to matched nontumorous pancreas duct: (i) H&E staining of pancreas tumor and adjacent nontumorous pancreas duct, (ii) IHC of nontumorous pancreas duct versus pancreatic tumor, (iii) a high power view of adjacent nontumorous pancreas duct, and (iv) A high power view of a pancreatic tumor. The blue arrows indicate the nontumorous pancreas ductal cells, and red arrows indicate pancreatic tumor cells.

The protein expression of LAMP1 and ORP150 was investigated by immunohistochemistry in eight pairs of pancreatic cancer and matched non-tumorous pancreas tissue sections. All pathological materials and the IHC results were reviewed and scored by an American Pathology Boards certified pathologist. Figure 4A showed that LAMP1 was over-expressed in tumor cells but not in the paired nontumorous pancreas ductal cells. Seven of eight pancreatic cancer cases were stained strongly (score ≥ +2) while no staining was detected in matched nontumorous pancreas tissues. A similar phenomenon was observed for ORP150. The expression of ORP150 in cancer cells was significantly increased as compared to adjacent tumor-matched normal pancreas ductal cells (Figure 4B). Numbers of no/weak staining and moderate/strong staining was compared between tumor cells and matched nontumorous pancreas ductal cells. Both LAMP1 and ORP150 showed over-expressions in pancreatic tumor cells (*P*-value = 0.0014).

## Discussion

Glycosylation is a complex co-/post-translational modification of proteins that mediates a variety of cell surface recognition and binding events, participates in many facets of the vertebrate immune response and contribute to the progression of many diseases including the focus of this study, which is cancer [56]. Glycans, however, have remained relatively poorly understood because of challenges in identifying glycoproteins, determining the occupancy status and precise oligosaccharide structures located at specific glycosylation sites due largely to the complexity of the glycosylation process [56]. In this study, we used metabolic oligosaccharide engineering methods to identify the sialoglycoproteins modified by azido-sugar in metabolically engineered pancreatic cancer cells when the cells were over-supplied with sialic acid substrate analog.

Pancreatic cancer is the fourth leading cause of death by cancer in Europe and in the US [57]. Among the common malignancies, pancreatic cancer has the highest mortalities with a 5-year survival rate of less than 5% [58,59]. Poor prognosis of pancreatic cancer is due to the aggressive nature of this disease, rapid metastasis, and late diagnosis of the malignancy [60]. Thus, identification of biomarkers as targets to diagnose and treat pancreatic cancer is urgently needed, which provides the impetus for our efforts to discover pancreatic cancer-associated sialoglycoproteins in the current report.

Because the heterogeneity of protein glycosylation is evidenced by a number of potential glycosylation sites on individual glycoprotein, different glycosylation sites may be modified by different carbohydrates, of which abundance may also differ from the protein expression level [61]. Compared to a previous strategy that used metabolic engineering to isolate sialoglycoproteins and associated proteins in the protein mixture [29], the major advantage of the approach used in this study is the capture of azido labeled sialoglycopeptides and enzymatic release of *N*-linked sialoglycopeptides for mass spectrometry identification of sialoglycosylation sites. The procedure allows the identification of not only the isolated proteins, but also the specific *N*-glycosylation sites that may contribute to more sensitive and specific identification of sialoglycoproteins [29]. Another advantage of our approach compared to previous reports is the use of synthetic azido-derivatized ManNAc analog 1,3,4-*O*-Bu_3_ManNAz, which is capable of supporting higher metabolic flux through the sialic acid biosynthetic pathway at low concentrations compared to Ac_4_ManNAz [29,34].

Seventy-five unique formerly azido-sialic acid modified glycopeptides were identified from 55 glycoproteins using 1,3,4-*O*-Bu_3_ManNAz analog treatment (Table 2 and Additional file 2: Table S2). The number of proteins we identified was relatively low compared to whole glycoproteome datasets, in which hundreds or even thousands of glycoproteins are expected to be expressed in a particular cell type. However we emphasize that we deliberately did not seek to maximize detection of all possible sialoglycans in the present study but rather conducted our experiments in a way that would be most conducive to the identification of cancer-associated biomarkers. In particular, we first limited the labeling period to two days even though evidence suggests that non-natural sialic acids can accumulate in tissue over a 6-week period upon repeated daily dosing [62]; we consider it to be unlikely that a diagnostic test could be conducted in a clinically-relevant manner over this extended period (instead we used a 2-day period in which a single bolus of analog has been shown to resist esterase degradation [63] and further, maximize cellular labeling without the need for added doses or replenishment). Second, the number of cells involved in each experiment (<10e7) coupled with the subfemtomolar mass spec detection sensitivity limit implies that only relatively abundant markers (e.g., glycoproteins expressed in the hundreds to thousands of copies per cell) will be detected by our methods; again, this feature is desired to ensure that our methods will have clinical relevance where sample size is likely to be limiting. Finally, as described in our previous publication [9], we use stringent statistical cutoffs to avoid false positives. Together, these factors – by design – limit the number of glycoproteins that are identified by our method and ensure that the glycoproteins we identify will be sufficiently abundant and metabolically labeled with sufficient kinetics to be viable cancer biomarkers.

Another matter of interest is the exact glycan structures that the non-natural azido-modified sialic acids label in cancer cells. In our previous experiments using 1,3,4-O-Bu_3_ManNAc, which increased levels of *natural* sialic acid by ~75% in treated cells [9], the levels of sialylation were sufficiently higher in the treated cells to allow characterization of the new glycoforms by using mass spectrometry-based methods [64]. By comparison, treatment of cells with non-natural analogs tends to result in the replacement of natural sialic acids with their non-natural counterparts rather than an overall *increase* in sialylation (for perspective on this issue, consult references [65-68]. In general, even though several million non-natural sialic acids can be incorporated on the surface of metabolically oligosaccharide engineered cancer cells [69], the number of these moieties constitute only a small percentage of the hundreds of millions of sialic acids typically found on each human cell [69]. Consequently, rigorous detection of non-natural sialic acids by unambiguous techniques such as mass spectrometry because of their low abundance remains extremely challenging and alternative approaches such as lectin binding assays have provided some insight.

Lectins have long been used for purification and detection of glycans in many studies [70]; many lectins have overlapping affinity while some others have unique specificity, allowing for the efficient detection of differential expression of glycan structure. In the current studies the use of a previously-reported lectin array [35] showed significant binding signal from fourteen lectins (*p* < 0.05), indicating α-1-6 core fucoses (PSA), Galβ1-4GlcNAc structures (DSA and PHAE), high mannose (NPA, GNA, and HHL) among the *N*-type glycans, and Galβ1-3GalNAc (Jacalin and MPA) and GalNAcβ1-4GlcNAc (WFA) for *O*-type glycans (Figure 3 and Table 1). This data provided rudimentary insight into the glycan structures compatible with azido-sialic acid incorporation but the most interesting aspect of these experiments was the lack of. lectin binding to native sialic acid despite the presence of the appropriate lectins (e.g., SNA) on the array and the plentiful display of sialic acids on SW1990 cells demonstrated in our previous experiments [9,64]. Possible explanations for this outcome include that 1) azido-sialic acid was not well recognized by, or had weak binding, to sialic acid specific lectins, such as SNA or that 2) the proteins modified by native sialic acid are not simultaneously modified by azido-sialic acid.

As a final part of this project, we return to the initial motivation of this study, which was to use metabolic oligosaccharide engineering methodology to identify cancer-associated biomarkers in pancreatic cancer. This task is complicated by not having a high quality “normal” pancreatic line that readily grows in cell culture conditions for comparison. Therefore, one way we attempted to address this issue was to cross-reference the list of the 55 proteins we identified against cancer (in general) and pancreatic cancer (more specifically) and found that 42 proteins have been reported to be associated with cancer in general and 15 have been specifically linked to pancreatic cancer (Additional file 2: Table S2). Although encouraging, the significance of this type of analysis is open to interpretation considering that many of the proteins are linked to cancer by as few as one reference, and further, it is not unexpected to detect cancer-related proteins by analyzing cancer cells. Therefore, we tried an alternative approach that we consider to be more stringent by analyzing clinical samples for two proteins (LAMP1 and ORP150) not previously associated with pancreatic cancer and found preferential expression in tumors compared to normal tissue in both cases (Figure 4). Although far from definitive (for example, comprehensive analysis of all the identified glycoproteins is beyond the scope of the current investigation), this early result strongly supports our metabolic oligosaccharide engineering approach as a viable strategy for the identification of new cancer biomarkers.

In conclusion, this paper describes a strategy to analyze metabolically active sialoglycoproteins found in pancreatic cancer cells and shows that robust labeling of several proteins already associated with tumors occurs. Even more significantly, the positive evaluation of two proteins not previously associated with pancreatic cancer in clinical samples suggests that this method may be a powerful technique for biomarker discovery. In summary, the results showed that metabolic oligosaccharide engineering had broader implications by the discovery of sialoglycoproteins in cancer cells that hold potential for the diagnosis of cancer or as cell surface therapeutic targets [71].

## Materials and methods

### Cell culture

The pancreatic cell line, SW1990, was purchased from the American Type Culture Collection (ATCC; Manasses, VA). Cells were grown in 1:1 DMEM medium supplemented with 10% (v/v) FBS and 1% (v/v) of a solution of 10,000 units penicillin and 10 mg streptomycin/mL, at 37°C and 5% CO_2_.

### Metabolic labeling of cells and cell surface azide visualization via fluorescence microscopy

Pancreatic cells were seeded on 6-well TC plates and synthetic 1,3,4-*O*-Bu_3_ManNAz analog [34] from a 50 mM stock solution was added to each well prior seeding the cells to provide a final concentration of 50 μM. Cells were incubated for two days with or without the analog. Prior to visualization, the cells were washed with PBS and fixed with 3.7% formaldehyde for 20 min at room temperature. Labeling was done by adding 200 μL of a freshly prepared Click Reaction Mixture to each well followed by incubation in the dark for 45 min as described previously [34]. The cells were then washed 3–5 times with PBS containing 5% BSA. Images were taken using a Zeiss Observer microscope with a 40X objective lens (Zeiss, Inc., Melville, NY). Fluorescence pictures of Alexa488 (surface sialoglycoconjugates), and DAPI (nucleus) labeled cells were recorded and overlay images were generated with the Zeiss Imaging System.

### Biotinylation of azido-sialic acid containing glycoproteins

To selectively biotinylate azido-sialic acid modified glycoproteins, the modified Staudinger ligation reaction reported by Saxon and coworkers using Biotin-PEG_3_-Phosphine was performed on proteins extracted from cell lysates [15]. The protein concentration was adjusted to 2.5 mg/mL in PBS (pH 7.2), Biotin-PEG_3_-Phosphine was added to a final concentration of 200 μM. The sample was incubated overnight at room temperature. The biotinylation efficiency and specificity was examined by Western blot using HRP-streptavidin at 1:5000. The non-reacted Biotin-PEG_3_-Phosphine was removed through protein precipitation. The proteins were mixed with an 8-fold volume of pre-chilled (−20°C) acetone by vortexing and then incubated at −20°C for 60 min. The samples were centrifuged at 13,000 × g for 10 min. The supernatant was removed and disposed without disturbing the protein pellet. This washing and precipitation process was repeated and residual acetone was allowed to evaporate from the uncapped tube at room temperature for 30 min.

### Affinity purification of biotinylated proteins

A 50% slurry of monomeric avidin agarose (1.4 mL) resin was sequentially washed with 3 mL PBS, 3 mL of 2 mM d-biotin in PBS, 3 mL of 0.1 M glycine (pH 2.8), and 3 mL PBS. Protein (500 μg per sample, isolated from treated or untreated cells as described above) was added to the pre-conditioned monomeric avidin agarose resin and incubated at room temperature for 1 h, followed by five washes with 6 mL PBS. The biotinylated azido-modified glycoproteins were eluted with 0.7 mL of 2 mM D-biotin in PBS for 10 min followed by a second elution step of 0.7 mL of 0.1 M glycine (pH 2.8) for 10 min.

### Characterized sialic acid glycans using lectin microarrays

A lectin microarray comprised of 38 lectins as described in our previous study was used to profile the glycan structures of the proteins biotinylated via Staudinger ligation [35]. Each lectin was prepared in three concentrations (1, 0.5, and 0.25 mg/mL) on the chip. The slides were blocked with 50 mM ethanolamine in 40 mM sodium borate for 1 h followed by three subsequent washes with PBS-0.1% Tween. The biotinylated proteins (100 μL) were applied to each lectin microarray. Inhibitory sugar solutions (e.g. 100 μL of 50 mM lactose, 50 mM galactose, 50 mM mannose, or 50 mM acetic acid) were mixed with samples, and then were applied to the control wells. After an overnight incubation at room temperature, each well was washed with 100 μL of PBS-0.1% Tween 20 with 5 min shaking. Rabbit IgG (20 μg) was added to each array followed by a 30 min incubation to mask any residual lectin sites. The slides were washed three times with PBS-0.1% Tween 20. Afterwards, 100 μL of streptavidin conjugated to Dylight 549 (diluted 500-fold in PBS-0.1% Tween) was added to each well and incubated for 30 min in the dark before the array was washed, dried, and scanned with a GenePix 4100B scanner (Sunnyvale, CA).

### Identification of formerly azide-sialic acid modified glycopeptides

After biotinylation and removal of the non-reacted Biotin-PEG_3_-Phosphine using protein precipitation, 1 mg of protein was denatured and reduced in 8 M urea, 10 mM TCEP, and 1% SDS in 200 mM Tris buffer at 37°C for 2 h [72]. Iodoacetamide (10 mM final concentration) was added to the sample and incubated for 30 min at RT in the dark. The solution was diluted 8-fold with 100 mM KH_2_PO4 (pH 8.0). The proteins were digested by trypsin (Promega, 1:50, w/w) at 37°C overnight with shaking. After digestion, the samples were centrifuged at 16,110 × g for 5 min to remove particulate matter. The peptides were cleaned by using a Sep-Pak C18 Vac cartridge (Waters) and resuspended in PBS. Six hundred microliters of 50% slurry of streptavidin agarose resin was washed with 1 mL PBS (pH 7.2) three times. Protease inhibitor (Pierce) was added to the peptides at a 1:1000 dilution prior to mixing peptides with pre-washed streptavidin agarose. The peptides were allowed to bind to streptavidin agarose at room temperature for 15 min. The unbound peptides were removed by centrifugation. The streptavidin agarose was washed with 1 mL PBS 6 times, then resuspended in 250 μL G7 buffer (New England Biolabs) and peptides containing N-linked glycosylation sites with metabolic labeled sialoglyans were released by PNGase F (New England Biolabs) [73-75]. The peptides were analyzed using a LTQ-Orbitrap velos (ThermoFisher, Waltham, MA) coupled with a 15 cm × 75 μm C18 column (5 μm particles with 100 angstrom pore size). The spectra were analyzed as described in our previous report [76]. Briefly, a nanoAquity UPLC at 300 nL/min with a 90 min linear acetonitrile gradient (from 5-32% B over 90 min; A = 0.1% formic acid in water, B = 0.1% formic acid in acetonitrile) was used. A top 10 data dependent MS/MS with exclusion for 20 s was set.

MS/MS spectra were searched with MASCOT (version 2.2.0) using Proteome Discoverer (version 1.0) (Thermo Fisher) against human subdatabase of NCBI Reference Sequence (RefSeq) (version 40, released at April 16, 2010) containing 29,704 sequences. For this database search, the precursor mass tolerance and fragment mass tolerance were set at 15 ppm and 0.05 Da respectively. Trypsin was specified as the protease. The fixed modification was set as carbamidomethylation (C), and other database-searching parameters were set as flexible modification as follows: deamidation (NQ) and oxidation (M). Semi-tryptic end and one missed cleavage site was allowed. The False Discovery Rate was set at 0.01 to eliminate low-probability protein identifications.

Identified proteins were classified according to their main biological processes, their molecular functions and their cellular component using “Blast2Go” software (www.blast2go.com) [38].

### Immunohistochemistry (IHC)

Formalin-fixed and paraffin-embedded tumor tissue was obtained from the Department of Pathology at Johns Hopkins Hospital, and was cut into 4-micron sections. Slides were de-paraffined and rehydrated before IHC study. Dako LSAB+ System-AP kit (Carpinteria, CA) used for IHC study according to the manufacturer’s instructions. Antigen-retrieval using 10 mM sodium citrate (pH 6.0) at 100°C for 20 min was performed prior to apply mouse monoclonal anti- human LAMP1 antibody at 1:150 dilution. The mouse monoclonal anti- human ORP150 antibody was applied to slides at 1:200 dilution without antigen retrieval. The primary antibodies were incubated overnight at 4°C. All pathological materials and IHC results were reviewed and evaluated by an American Pathology Boards certified pathologist, Dr. Qing Kay Li. The cytoplasmic immuno-staining pattern and immuno-reactive intensity were scored semi-quantitatively using a 4 tier system: no staining (0), weak (1+, <10% positivity), moderate (2+, 10-50% positivity), and strong (3+, >50% positivity). The IHC staining pattern was correlated with tumors’ pathological stages and the differentiations. Patients’ clinical information was also correlated. Fisher’s exact test was used to determine the statistical significance of IHC staining in pancreas tumor and paired normal pancreas tissues.

## Additional files

Additional file 1: Table S1. Information of 38 lectins used in lectin microarray. Additional file 2: Table S2. Identification of N-glycosite-containing sialoglycopeptides using LC-MS/MS. Additional file 3: Table S3. MS/MS annotation for the identified sialoglycopeptides.
